# Transcriptomic Profiling Reveals Extraordinary Diversity of Venom Peptides in Unexplored Predatory Gastropods of the Genus *Clavus*

**DOI:** 10.1093/gbe/evaa083

**Published:** 2020-04-23

**Authors:** Aiping Lu, Maren Watkins, Qing Li, Samuel D Robinson, Gisela P Concepcion, Mark Yandell, Zhiping Weng, Baldomero M Olivera, Helena Safavi-Hemami, Alexander E Fedosov

**Affiliations:** e1 Department of Central Laboratory, Shanghai Tenth People’s Hospital of Tongji University, School of Life Sciences and Technology, Tongji University, Shanghai, China; e2 Department of Biology, University of Utah; e3 Eccles Institute of Human Genetics, University of Utah; e4 High-Throughput Genomics and Bioinformatic Analysis Shared Resource, Huntsman Cancer Institute, University of Utah; e5 Marine Science Institute, University of the Philippines-Diliman, Quezon City, Philippines; e6 Utah Center for Genetic Discovery, University of Utah; e7 Program in Bioinformatics and Integrative Biology, University of Massachusetts Medical School; e8 Department of Biochemistry, University of Utah; e9 Department of Biology, University of Copenhagen, Denmark; e10 A.N. Severtsov Institute of Ecology and Evolution, Russian Academy of Science, Moscow, Russia

**Keywords:** Conoidea, venom, *Clavus*, drillipeptides, venom gland, transcriptome

## Abstract

Predatory gastropods of the superfamily Conoidea number over 12,000 living species. The evolutionary success of this lineage can be explained by the ability of conoideans to produce complex venoms for hunting, defense, and competitive interactions. Whereas venoms of cone snails (family Conidae) have become increasingly well studied, the venoms of most other conoidean lineages remain largely uncharacterized. In the present study, we present the venom gland transcriptomes of two species of the genus *Clavus* that belong to the family Drilliidae. Venom gland transcriptomes of two specimens of *Clavus canalicularis* and two specimens of *Clavus davidgilmouri* were analyzed, leading to the identification of a total of 1,176 putative venom peptide toxins (drillipeptides). Based on the combined evidence of secretion signal sequence identity, entire precursor similarity search (BLAST), and the orthology inference, putative *Clavus* toxins were assigned to 158 different gene families. The majority of identified transcripts comprise signal, pro-, mature peptide, and post-regions, with a typically short (<50 amino acids) and cysteine-rich mature peptide region. Thus, drillipeptides are structurally similar to conotoxins. However, convincing homology with known groups of *Conus* toxins was only detected for very few toxin families. Among these are *Clavus* counterparts of *Conus* venom insulins (drillinsulins), porins (drilliporins), and highly diversified lectins (drillilectins). The short size of most drillipeptides and structural similarity to conotoxins were unexpected, given that most related conoidean gastropod families (Terebridae and Turridae) possess longer mature peptide regions. Our findings indicate that, similar to conotoxins, drillipeptides may represent a valuable resource for future pharmacological exploration.

## Introduction

The predatory gastropods of the superfamily Conoidea J. Fleming, 1822 are a megadiverse taxon of marine mollusks, whose rapid evolutionary radiation was made possible by the development of a complex venom apparatus in ancestors of recent conoideans (Bouchet et al. 2011; Abdelkrim et al. 2018). Conoidean venoms are complex and contain potent and often highly selective peptides that are used for prey capture and defense. It is widely accepted that the diversification of Conoidea was underpinned by evolution of both their venoms and venom delivery systems (Shimek and Kohn, 1981; Olivera et al. 1985; Taylor et al. 1993; Dutertre et al. 2014). The elegant, uniquely efficient envenomation mechanism of many conoideans, wherein the venom is injected into prey through a hollow hypodermic harpoon—a highly specialized type of the molluskan grazing organ, the radula—is best known from fish-hunting cone snails in the genus *Conus* Linnaeus, 1758 (Kantor and Taylor 2000; Olivera et al. 2015). The stings of some *Conus* capable of subduing and killing a vertebrate prey or predator can be deadly to humans (Kohn 2018). The discovery of greatly diversified, short peptides in *Conus* venoms that predominantly target ion channels and receptors in the nervous system with unprecedented selectivity (Olivera et al. 1985; Terlau and Olivera 2004) has led to growing multidisciplinary interest in these venoms. To date, venom peptides from over 300 species of cone snails have been characterized using various approaches (Puillandre et al. 2016; Phuong and Mahardika 2018; Phuong et al. 2019), establishing *Conus* venoms as an excellent model system for studying molecular evolution in venomous marine animals. Furthermore, given their unique diversity and selectivity profile, cone snail venom peptides have become indispensable pharmacological probes and have been developed as drugs and drug leads for pain, epilepsy, diabetes, and stroke (Jimenez et al. 2002; Prashanth et al. 2014; Balsara et al. 2015; Safavi-Hemami et al. 2019; Robinson and Safavi-Hemami 2017).

Although cone snail venoms have become a focus of intensive pharmacological studies, present knowledge of the venoms of conoideans outside the family Conidae has lagged far behind (Gorson et al. 2015; Puillandre et al. 2016). Given the vast diversity of over 10,000 estimated nonconid conoidean species (compared with ∼900 species of Conidae) venom composition reports in the relatively well studied genus *Conus* may merely be scratching the surface of the vast diversity of venom compounds evolved in the superfamily Conoidea (Abdelkrim et al. 2018).

This is in part a result of methodological difficulties—most conoideans are small snails (Bouchet et al. 2009), with sometimes exceedingly small venom glands, which cannot yield a sufficient amount of venom for biochemical characterization. However, the recent advent of deep sequencing not only provides a methodological basis for generating high quality data from miniscule amounts of venom but also provides the opportunity to efficiently address questions of venom composition and evolution of venom gene families in a single study. Therefore, it is to be anticipated that our understanding of venom evolution across the conoidean tree of life will improve drastically in the upcoming years, allowing for the harnessing of the vast pharmacological potential of the superfamily Conoidea. The first transcriptomic analyses of the conoidean families Turridae, Pseudomelatomidae (Gonzales and Saloma 2014), and Terebridae (Gorson et al. 2015), gave a glimpse of the venom composition in the conoidean lineages divergent from Conidae, but currently available data remain too fragmentary to attempt comparative analysis across Conoidea.

**Fig. 1.**
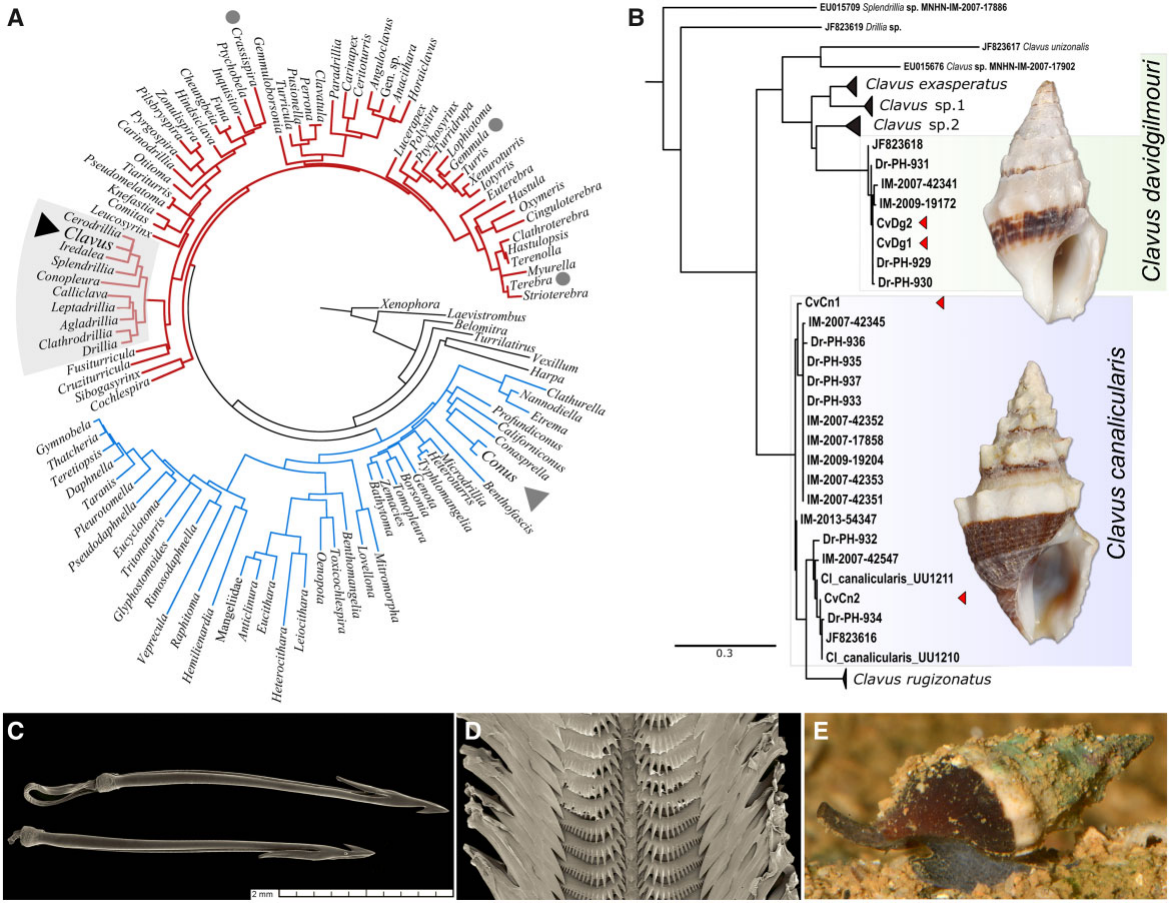
—Phylogeny and morphology of the genus *Clavus*. (*A*) Phylogenetic tree of the superfamily Conoidea (after Puillandre et al. 2016). (*B*) Relationships of the Philippine species of the *Clavus canalicularis* species complex. (*C*) Marginal radular teeth of *Conus* (*Pionoconus*) *circumcisus* representing a hypodermic type. (*D*) Radula of *Cv. canalicularis* with distinct comb-like lateral teeth. (*E*) Live *Cv. canalicularis* (photo—courtesy of David Massemin).

In this study, we present a comprehensive survey of the venom gland transcriptomes of two species of the genus *Clavus* Montfort, 1810, which belongs to the family Drilliidae Olsson, 1964, one of the major conoidean families whose venoms remain uncharacterized. Its members retain a set of plesiomorphic characters in morphology, which led many authors to place Drilliidae at the base of conoidean radiation (Taylor et al. 1993). Nevertheless, with about 530 living species currently classified to it (MolluscaBase, http://www.molluscabase.org/aphia.php? p=taxdetails&id=23032, last accessed April 26, 2020), this family is among the highly diversified lineages of Conoidea as well (Abdelkrim 2018) (fig. 1*A*). The unique structure of drilliid radulae implies a principally different mechanism of prey envenomation (Sysoev and Kantor 1989). Nevertheless, there is no published data on the diet and feeding biology of *Clavus* (and Drillidae in general). Transcriptomic profiling reveals a vast diversity of the putative *Clavus* venom components that we term “drillipeptides.” Although biological activity of these components is still to be established, their number in the transcriptomic data set notably exceeds the toxin diversity observed in individual specimens of *Conus* analyzed employing the similar methodology. The complement of putative toxins observed in individual specimens of *Clavus*, while closely parallel to *Conus* venoms in many respects, is clearly unique among Conoidea, most notably in the diversity of toxins found in each species analyzed.

## Materials and Methods

### Specimen Collection

All studied specimens were collected in the proximity of Olango Island (Central Philippines) on August 5, 2015. Specimens of *Clavus canalicularis* (Röding, 1798) and *Clavus davidgilmouri* (Fedosov and Puillandre forthcoming) (referred to as CvCn1 and CvCn2 for *Clavus canalicularis* specimen 1 and 2, respectively, and CvDg1 and CvDg2 for *Clavus davidgilmouri* specimen 1 and 2, respectively) were recovered from the same habitat—coral rubble at depths of ∼15–25 m by local fishermen and delivered to the laboratory alive on the same day, or the day after they were collected. After breaking the shell, the mantle was removed, and the body hemocoel dissected to extract the venom gland. Dissected venom glands were immediately suspended in *RNAlater* (Thermo Fisher Scientific). A tissue clip was taken from each dissected specimen and preserved in *RNAlater* for subsequent DNA extraction to confirm initial species identification. Samples in *RNAlater* were kept at room temperature for 24 h, and then frozen and kept at −20 °C until RNA extraction.

### DNA Extraction, Amplification, and Phylogenetic Analysis

To confirm identity and to ensure conspecificity of the specimens dissected for transcriptomic study, a barcode fragment of mitochondrial gene cytochrome oxidase I (COI) was sequenced for the four dissected specimens of *Clavus*, as well as for nine additional specimens. Total genomic DNA was extracted from foot muscle tissue using Thermo Scientific Gene-JET Genomic DNA Purification 50 Kit following the manufacturer’s instructions. Fragments of the COI gene were amplified with the standard pair of primers LCO1490 and HCO2198 (Folmer et al. 1994). Polymerase chain reactions (PCRs) were performed in 20 µl final volume containing ∼3-ng template DNA, 1.5 mM MgCl_2_, 0.26 mM of each nucleotide, 0.3 µl of each primer, 5% Dimethyl sulfoxide, and either 0.75 µl of Taq Polymerase (Qbiogene) or BioHYTaq DNA polymerase (Dialat, Moscow). The PCR profile for the COI started with 5 min at 95 °C followed by 40 cycles with the denaturation at 95 °C (35 s), annealing at 50 °C (35 s), and elongation at 72 °C (1 min), with final elongation phase at 72 °C (7 min). The amplified DNA fragments were sequenced in both directions to confirm accuracy of each sequence. The sequencing was performed in the SIEE RAS molecular facility on an ABI 3500 Genetic analyzer. Alternatively, the purified PCR products encoding *Clavus* COI sequences were annealed to a plasmid vector and transformed into competent cells as previously described (Fedosov et al. 2011). The nucleic acid sequences of six individual COI-encoding clones from each specimen were determined according to the standard protocol for automated sequencing at the Health Sciences Center Core Sequencing Facility, University of Utah, and compared to ensure sequence consensus.

A total of 39 specimens were included in the phylogenetic data set, including three species of the conoidean family Horaiclavidae as outgroups (taxonomy of the Conoidea after Bouchet et al. 2011). Of the 36 Drilliidae specimens analyzed, 17 belonged to the genus *Clavus*, including 13 specimens sequenced in the present study, and 4 records recovered from the GenBank sequencing database; all specimens originated from the Central Philippines. Sequences were aligned using ClustalW, as implemented in BioEdit 7.0.9.0 (Hall 1999). The 658 nucleotide positions in the COI alignment were treated as three independent partitions, corresponding to the three codon positions in the RaxML analysis (Stamatakis 2006). GTR + I + G was estimated as best fitting nucleotide substitution model by Modelgenerator v.85 (Keane et al. 2006) under Akaike information criterion. Robustness of nodes was assessed using the Thorough Bootstrapping algorithm (Felsenstein, 1985) with 1,000 iterations. Phylogenetic analysis was performed on the Cipres Science Gateway (http://www.phylo.org/portal2, last accessed April 26, 2020), using RAxML-HPC2 on XSEDE (Miller et al. 2010). The matrix of pairwise genetic distances for the COI alignment was calculated using MEGA 6 (Tamura et al. 2013).

### RNA Extraction and Sequencing

For transcriptome sequencing, total RNA extraction from venom glands was performed using the Direct-zol RNA extraction kit (Zymo Research, CA), with on-column DNase treatment, according to the manufacturer’s instructions. Venom glands were homogenized in Direct-zol TRI reagent using the TissueRuptor II (Qiagen) with a disposable probes to avoid sample cross-contamination. cDNA library preparation and sequencing were performed by the University of Utah High-Throughput Genomics Core Facility. Briefly, total RNA quality and quantity were first determined on an Agilent 2200 TapeStation (Agilent Technologies). A dual-indexed library was constructed with the Illumina TruSeq Stranded mRNA Sample Prep Kit with oligo (dT) selection and an average insert size of ∼220 bp. The library was validated on an Agilent 2200 TapeStation and using a quantitative PCR assay (KAPA Biosystems Library Quantification Kit for Illumina). The 125-cycle paired-end sequencing was performed on an Illumina HiSeq2000 instrument (San Diego, CA). The statistics of the obtained raw reads data are summarized in supplementary table 1, Supplementary Material online.

### Transcriptome Assembly

Each set of paired-end raw reads was first interleaved and processed by FQTRIM (version 0.9.4, http://ccb.jhu.edu/software/fqtrim/, last accessed April 26, 2020) and prinseq-lite (version 0.20.4) (Schmieder and Edwards 2011) to remove adapter and low quality base pairs or reads. Bases at the 3′-end were trimmed until the quality score was above 20, and polyA/T tails in good quality were kept. Reads with at least one base quality score below 10 or <80 bp in length were discarded. RNA reads remaining after quality control were error corrected (each set of reads separately), and then assembled using TRINITY v2.2.0 (Haas et al. 2013) with kmer size used to build the De Bruijn Graphs set to 31 (kmer size was optimized for *Conus* transcriptome assembly as described by Li et al. [2017]). The assembly metrics for the four specimens of *Clavus* are summarized in the supplementary table 2, Supplementary Material online.

### Transcript Annotation

Transcriptome assemblies were annotated using NCBI-BlastX against a combined ConoServer (Kaas et al. 2012) and UniProtKB database (release April 2015, BlastP *e*-value < 10e-10). The conotoxin sequences published in 2015–2016 that were not yet uploaded into UniProt at the time of the analysis (Barghi et al. 2015a, 2015b; Phuong et al. 2016) were also added to our in-house database. Transcript contigs that did not have significant hits in known databases were translated into six frames, and predicted open reading frames (containing a start and stop codon) with a total length more than 50 amino acids (AAs) were retained for further analysis. Subsequently, the predicted sequences were analyzed, and only those with N-terminal signal peptides (predicted using SignalP [Petersen et al. 2011], *D* value > 0.7) but no transmembrane topology (predicted using Phobius [Käll et al. 2007]) were kept. Transcriptome assembly software programs are known to generate misassembled contigs, particularly for sequences with low read coverage. To address this, we only report transcripts that were identified in at least two specimens and with tpm values >100. Prior *Conus* transcriptome studies have demonstrated that using assembly software alone (e.g., Trinity) does not reveal the full diversity of transcripts, particularly for those deriving from polymorphic loci (Phuong et al. 2016; Robinson et al. 2017; Macrander et al. 2018). Remapping of trimmed reads onto assembled sequences can partially retrieve these otherwise evasive sequences (Robinson et al. 2017; Macrander et al. 2018). However, this has to be done semimanually and, in our hands, is too subjective to be reproducible. We therefore only report contigs assembled by Trinity but would like to emphasize that this methodology might not fully retrieve the diversity of venom peptide in *Clavus*.

Many transcriptomic analyses of the genus *Conus* assume varying degrees of signal sequence identity (Barghi et al. 2015a; Peng et al. 2016), which reflect a priori knowledge of signal sequence conservation among toxin gene superfamilies. The venom composition of the conoidean family Drilliidae, however, remains terra incognita, and it is not only that the number of toxin gene superfamilies expressed in the venom is unknown but also that there is no information regarding the level of conservation of the signal sequences in relation to the mature peptide regions. This hampers inference of the common origin of the predicted venom peptides, which is necessary to delineate gene superfamilies. In particular, it is unclear whether the gene superfamilies in *Clavus* venom can be established based on comparable degrees of sequence identities as in *Conus*. To tackle this issue, we generated alternative clustering schemes based on different signal sequence identity thresholds, and then rated them to select an optimal scheme that would define putative gene superfamilies. To infer clusters of *Clavus* venom gland transcripts based on the similarity of their signal sequences (Puillandre et al. 2012), we used CD-Hit (Huang et al. 2010) with percent sequence identity threshold values of 75, 70, and 65. However, even the 65 identity threshold resulted in a very high number of putative gene superfamilies (>180), which would complicate comparisons with conotoxins. Therefore, we further reduced the percent signal sequence identity values to 60%, 55%, and 51%.

The drawback of the CD-Hit is that there is no clear measure to compare alternative cluster sets and select the one that best fits the data set. To rate the six obtained clustering schemes, we used two approaches. First, we estimated the level of structural heterogeneity of the mature peptide region based on the number of Cys-patterns in the inferred clusters (as the cysteine framework is an important determinant of the physiological activity of *Conus* toxins and is used for toxin classification—Terlau and Olivera 2004). Second, we classified inferred transcripts based on 1) BLAST hits of the entire precursor against the external databases (i.e., *Clavus–Clavus* hits, and *no BlastX hit* entries were removed from the data set) and 2) sets of orthogroups recovered for the entire precursor by OrthoFinder 2.1.2. (also see below). The 105 BLAST hits were classified in 23 clusters, and 206 orthogroups with two or more sequences in each were returned by OrthoFinder. Subsequently, an in-house Python script that implements a pair-counting approach (Wagner S and Wagner D 2007) was used to estimate how well each of the proposed signal-sequence-based cluster sets matches the BLAST-based cluster set. As the identified orthogroups are certainly more conserved, another Python script has been used to calculate how many of the identified orthogroups were split between two or more putative gene superfamilies. These numbers were used as penalty scores: the lower the score, the closer the match between the signal-sequence-based clusters and entire precursor-based orthogroups. The signal-sequence-based set of clusters that returned the highest score in comparison with BLAST clusters and lowest penalty in comparison with orthogroups was selected for putative gene superfamily breakdown.

All-by-all NCBI-BlastN searches were carried out to identify reciprocal best-hit (rbh) pairs among the inter/intra-specific drillipeptide nucleotide sequences. ClustalW alignment was then used to globally align the rbh pairs (Larkin et al. 2007). The alignment results were parsed to obtain the percent identity of each aligned rbh pair. To investigate interspecific differences at the sequence level, we performed comparative sequence analyses between the putative venom repertoires of each species (combined *Cv. canalicularis* data set vs. combined *Cv. davidgilmouri* data set). All-by-all NCBI-BlastN searches identified 177 and 165 rbh pairs between the two specimens of each *Clavus* species (*Cv. canalicularis* and *Cv. davidgilmouri*, respectively) and 209 rbh pairs when comparing the two species to one another (fig. 3).

To detect orthogroups in the *Cv. canalicularis* and *Cv. davidgilmouri* transcriptomes, we used OrthoFinder 2.1.2 (Emms and Kelly 2015) with the two input files corresponding to combined *Cv. canalicularis* and combined *Cv. davidgilmouri* data sets, with default settings. Separately for the l-Rhamnose-binding lectins (l-RBLs) and insulins, the low TPM transcripts were also recovered from the assembly and added to the data set. To calculate correlation between the *Clavus* species in transcript diversity and expression levels, Pearson’s *R* was calculated using the numpy method (numpy.corrcoef).

### Structure Prediction of Toxin Precursors

Conotoxin precursors are typically composed of an N-terminal signal peptide, a mature peptide region, and an intervening propeptide region between the signal peptide and mature peptide (e.g., Terlau and Olivera 2004). Some precursors also contain a postpeptide C-terminally of the mature peptide. For the *Clavus* data set, the signal peptide was characterized using SignalP 4.0 server (Petersen et al. 2011). Although the exact boundary of the mature peptide is difficult to assign without peptide level confirmation due to possible alternative cleavage events (Lu et al. 2014), a canonical mature peptide region was still predicted for each transcript in order to analyze *drillipeptide* precursor structure. The *Clavus* cysteine-rich peptide precursors were first predicted by an custom PERL script and then confirmed using ConoServer (Kaas et al. 2012) and ProP 1.0 server (Duckert et al. 2004). In peptide precursors containing no cysteine residues, the mature peptide regions were mainly predicted according to sequence alignment to known similar sequences. The custom PERL script to facilitate canonical mature peptide identification was designed to predict cleavage sites based on the published data (Fan and Nagle 1996; Fan et al. 2000; Duckert et al. 2004; Kaas et al. 2012). Briefly, the N-terminal boundary of the mature peptide was first identified C-terminally of two consecutive basic AAs (RR-, KR-, RK-, and KK-) before and closest to the first cysteine residue after removing the signal peptide from the precursor. If a single basic AA remained between the N-terminus and the first cysteine, and the distance to N-terminus exceeded five AAs, C-terminal of this basic AA was considered as the N-terminal boundary of the mature peptide. The C-terminal boundary of the mature peptide was identified by the presence of peptidylglycine α-amidating monooxygenase enzyme recognition sites consisting of a glycine followed by a basic or dibasic residue (-GKR-, -GRR-, -GRK- -GK-, -GR-, -KR-, and -RR-) at the C-terminal part of the last cysteine residue.

## Results and Discussion

### Phylogenetic Analysis Identifies Two Studied Species as Close Relatives

The phylogenetic analysis based on the barcode fragment of COI placed the dissected *Clavus* specimens in two different well supported clades, corresponding to *Cv. canalicularis* and *Cv. davidgilmouri* (fig. 1*B*). Although the *Cv. davidgilmouri* clade appeared to be homogeneous (highest within-clade K2P of 0.7%), the *Cv. canalicularis* clade showed clear genetic structure, with the highest within-clade genetic distance of 3.4%. The two *Cv. canalicularis* specimens studied for the venom gland transcriptome, although collected from same locality, showed a genetic distance of 2.7%, which is notably higher than between the two studied specimens of *Cv. davidgilmouri*. Nevertheless, the inferred pairwise comparisons within each clade fall within a range of intraspecific genetic distances demonstrated for the superfamily Conoidea (Puillandre et al. 2010; Fedosov and Puillandre 2012; Fedosov et al. 2017). The mean between-clades K2P genetic distance is 8.1%, and smallest—6.9%, which indicates that the two species, although closely related, are not sister species consistent with the previous results of Fedosov et al. (in preparation).

### *Clavus* Putative Toxin Diversity Is Unparalleled among Conoidea

A total of 1,176 unique putative toxin-like peptides were identified in the combined *Clavus* data set. They constitute 1.85% of the number of unique contigs recovered, and account for 28.8% of the total gene expression level (calculated from tpm values).

Typically, the identified transcripts demonstrated a canonical organization with a 16–34 AA signal sequence, followed by a pro-region of varying length (2–263 AA residue, lacking in ∼28% of identified putative precursors), a mature peptide region and, in ∼30% of identified transcripts, a short, one or few AA post region.

The number of clusters recovered by CD-Hit for the signal sequences of the putative *Clavus* toxins ranged from 120 (sequence identity threshold 51%) to 244 (sequence identity threshold 75%). In all clustering schemes, the number of Cys-patterns per cluster ranged from one to nine, and the proportion of clusters with only one Cys-pattern ranged from 0.55 to 0.82 when the sequence identity threshold was set at 51% and at 75%, respectively (supplementary fig. 2, Supplementary Material online). The CD-Hit clusters recovered when the signal sequence identity was set at 60 most closely match the clusters inferred by BLAST and OrthoFinder (supplementary table 3, Supplementary Material online), and therefore, they were used to designate the putative *Clavus* venom gene superfamilies. The resulting partitioning scheme includes as many as 158 putative gene superfamilies, each comprising 1–110 predicted toxins. At present, we propose naming them by prepending a superfamily number by the term “*Drillipeptide*,” however, more elaborated or convenient nomenclature may be developed in the future, as our knowledge of drillipeptides grows. Members of only 39 gene superfamilies had external BLAST Hits. From here onward, we will refer to the venom peptides that demonstrate sequence similarity to known groups of *Conus* toxins as A-like drillipeptides, F-like drillipeptide, etc. or drilliporins, drillinsulins, drillidipines, drillikunitzins. However, we emphasize that this often remote sequence similarity does not necessarily imply sequence homology.

Among the putative toxins with BLAST hits, the “MWIRK”-superfamily-like transcripts showed highest diversity and total expression level. Interestingly, each of the three highly expressed and diverse groups of peptides in *Clavus* venom, the “MWIRK”-superfamily-like transcripts, drilliporins, and l-RBLs, was represented by several gene superfamilies as defined by the signal sequence (four, three, and four, respectively). Conversely, only one drillipeptide gene superfamily produced BLAST hits to each of conotoxin superfamilies A, F, I3, M, O1, O2, U, *Conus* insulins, conodipine, and conkunitzin.

The high diversity and expression levels of the “MWIRK”-superfamily-like transcripts, drilliporins, and l-RBLs in the *Clavus* venom gland transcriptomes suggest that they play an important role in prey capture or defense (see below). Nevertheless, additional data on the spectrum of biological interaction of *Clavus* spp. are needed to pose these data in ecological context. The fact that in some cases, transcripts with similar BLAST hits represent different gene superfamilies may be explained by either: 1) the signal sequence being less highly conserved compared with typical conoidean gene superfamilies or 2) putative toxins with similar mature peptides having been recruited from different genes, or finally 3) the reference sequences of conoporins and l-RBLs actually representing heterogeneous groups themselves.

The Shannon’s index for transcript diversity was slightly higher in specimens of *Cv. davidgilmouri* (4.47 and 4. 32) compared with *Cv. canalicularis* (4.24 and 4.26) (supplementary table 6, Supplementary Material online). A recent transcriptomic study on two closely related species of *Conus*, *Conus tribblei* and *C*. *lenavati* (Barghi et al. 2015b), reported the highest toxin diversity of any cone snails characterized to date (Shannon’s index 3.3 in both species). Shannon’s index values in both species of *Clavus* exceed those in *C. tribblei* and *C. lenavati*, whereas the Shannon’s evenness values were comparable and ranged from 0.9 to 0.93 and from 0.89 to 0.92 in the *C. tribblei*–*C*. *lenavati* and among *Clavus* species, respectively. Although we believe that the vast majority of transcripts identified in this study are likely to be part of the toxin repertoire of *Clavus*, we cannot rule out that a small subset of transcripts is not used in predation but as endogenous signaling peptides or falsely predicted to be translated into a secreted peptide product. Future proteomic studies are necessary to verify predicted venom peptides of *Clavus*, whereas functional evaluations will be necessary to unambiguously distinguish toxins from endogenous signaling peptides.

### Putative *Clavus* Venom Repertoires Are Conserved at Gene Superfamily Level, and Highly Divergent at Mature Peptide

**Fig. 2.**
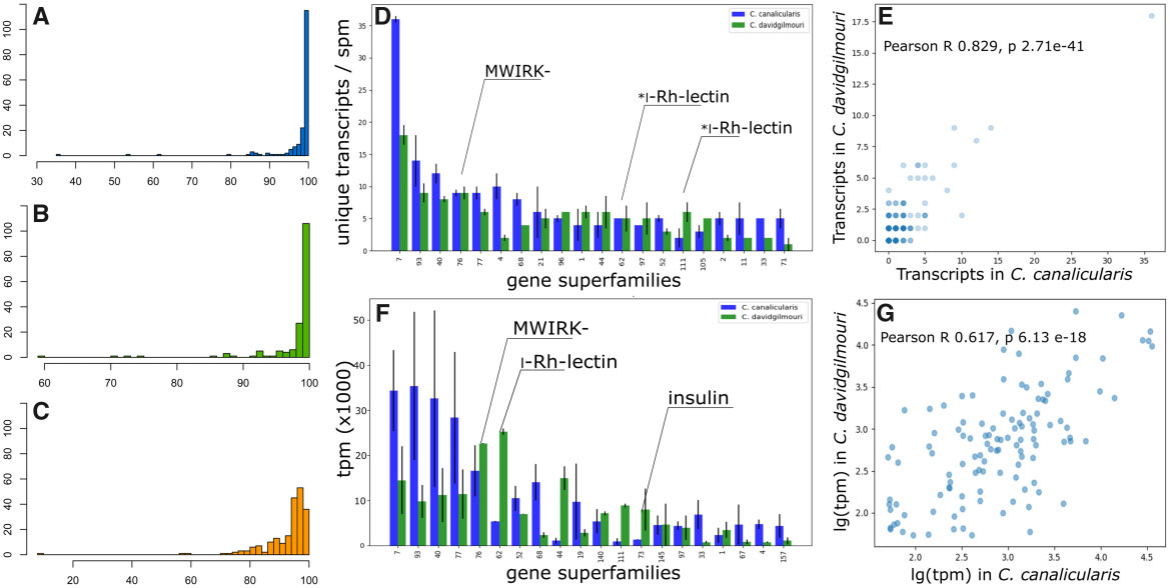
—Overview of *Clavus* spp. venom gland transcriptomes. (*A*–*C*) RBH analysis between conspecific specimens and the two species of *Clavus*. OX, percent identity; OY, RBH pair count. (*A*) *Cv. canalicularis* 1 versus *Cv. canalicularis* 2. (*B*) *Cv. davidgilmouri* 1 versus *Cv. davidgilmouri* 2. (*C*) *Cv. canalicularis* versus *Cv. davidgilmouri*. (*D*) Bar graph of the putative toxin gene superfamilies counts in the *Cv. canalicularis* and *Cv. davidgilmouri* (error bars denote standard deviation, spm = specimen). (*E*) Scatterplot of the putative toxin gene superfamilies counts in the *Cv. canalicularis* (*x*) and *Cv. davidgilmouri* (*y*). (*F*) Bar graph of the putative toxin gene superfamilies total expression levels in the *Cv. canalicularis* and *Cv. davidgilmouri* (error bars denote standard deviation). (*G*) Scatterplot of the putative toxin gene superfamilies total expression levels (log-normalized) in the *Cv. canalicularis* (*x*) and *Cv. davidgilmouri* (*y*).

In total, 644 and 532 unique transcripts were identified in the transcriptome of the venom glands of *Cv. canalicularis* and *Cv. davidgilmouri*, respectively. Comparative analysis of the putative toxin sequences between the two *Cv. canalicularis* specimens revealed that 89.3% of rbh pairs were >95% identical, and of these, 65.0% of sequences were >99% identical (fig. 2*A*). In the two specimens of *Cv. Davidgilmouri*, 89.7% of rbh pairs were >95% identical, and of these, 64.2% of sequences were >99% identical (fig. 2*B*). Finally, the comparative analysis of the combined *Cv. canalicularis* and *Cv. davidgilmouri* data sets revealed that 55.5% of putative toxin precursors shared >95% identity, but of these only 6.2% of sequences were more than 99% identical (fig. 2*C*). These data indicate that despite high overall similarity between the venom gland transcriptomes of the two *Clavus* species, considerable variation exists at the individual sequence level.

The most diverse gene superfamilies (7, 93, 40, 76, and 77) were represented in each *Clavus* species by 13 or more unique putative toxins and account for almost 25% of the total diversity of the identified transcripts (fig. 2*D*). Overall, a moderately strong correlation in the number of unique sequences per gene superfamily was observed both between the two species of *Clavus* (Pearson’s *R*, 0.83), and between the two studied specimens of each species (Pearson’s *R* 0.89 and 0.85 for the *Cv. canalicularis* and *Cv. davidgilmouri*, respectively) (fig. 2*E* and supplementary table 5, Supplementary Material online). The slightly weaker correlation between the two specimens of *Cv. davidgilmouri* is probably a result of different assembly statistics, with twice as many contigs identified in CvDg2 compared with CvDg1. Conversely, when expression levels of the gene superfamilies are compared (fig. 2*F* and *G*), considerable interspecific variation can be noted in most gene superfamilies. Furthermore, the gene superfamily expression levels vary greatly among the specimens of *Cv. canalicularis* compared with specimens of *Cv. davidgilmouri* (Pearson’s *R* 0.62 and 0.85 for the *Cv. canalicularis* and *Cv. davidgilmouri*, respectively). The latter result cannot be attributed to artifacts of assembly (the assembly statistics are very similar between the two *Cv. canalicularis* specimens whose transcriptomes were analyzed). It can be, however, explained by the notably higher genetic divergence between the two *Cv. canalicularis* specimens, inferred from the COI sequences, suggesting that the profile of the venom gene family expression is highly volatile and may vary considerably among conspecific individuals.

Nevertheless, the results of the RBH analysis in *Clavus* suggest that the repertoire of putative toxin gene families expressed in venom gland remains largely conserved within species and shows divergence between the closely related, (but not sister) species. This result closely parallels intra- and inter-specific comparisons reported in two closely related sister species of *Conus*; *Conus praecellens* and *Conus andremenezi* (Li et al. 2017) that showed a similar degree of genetic differentiation compared with the *Clavus* species studied herein. The generally strong correlation in the number of unique transcripts per gene superfamily between *Cv. canalicularis* and *Cv. davidgilmouri* corroborates this pattern, implying similar degrees of molecular diversity across the main putative venom components in the two species. Finally, the observed high variation in the putative venom gene families in *Cv. canalicularis* may be explained by the notable genetic divergence between the two dissected specimens (fig. 1*B*). Although a more rigorous estimate of the venom variation in *Clavus* is needed to validate and more accurately quantify the observed variation and disentangle its correlates, our results are consistent with gene superfamily expression being the most variable parameter of venom composition. Being fine-tuned to niche parameters, venom gene expression may profoundly change starting at early stages of the speciation process. Both the profile of molecular diversity (assessed through the number of unique gene products per venom gene superfamily) and the venom composition at the sequence level appear to be more conserved and are expected to contribute to venom variation at a higher taxonomic level.

### *Clavus* Venom Peptides Are Comparable to Conotoxins in Sequence Length and Structural Properties

**Fig. 3.**
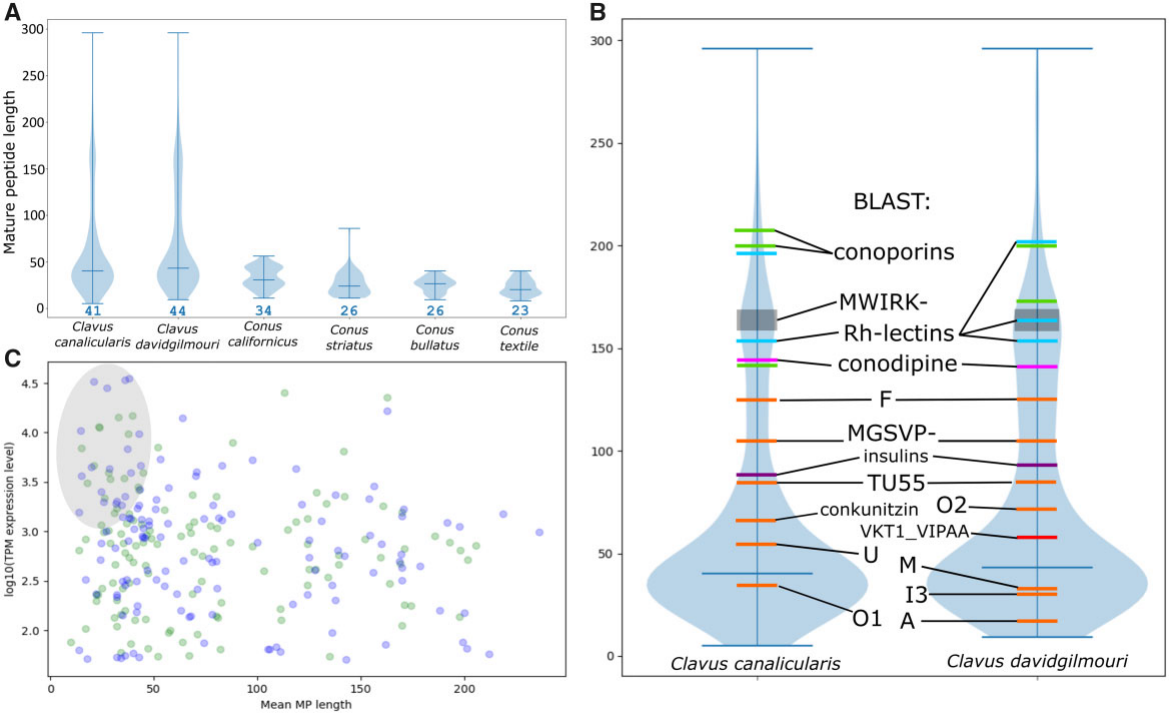
—Mature peptide region length distribution. (*A*) Violin plot of mature peptide region size in *Conus* and *Clavus* spp. (*B*) Mature peptide lengths in the putative *Clavus* toxins with BLAST hits. (*C*) Scatterplot of putative *Clavus* toxin gene superfamilies—mature peptide region length versus log 10 normalized tpm expression level.

**Fig. 4.**
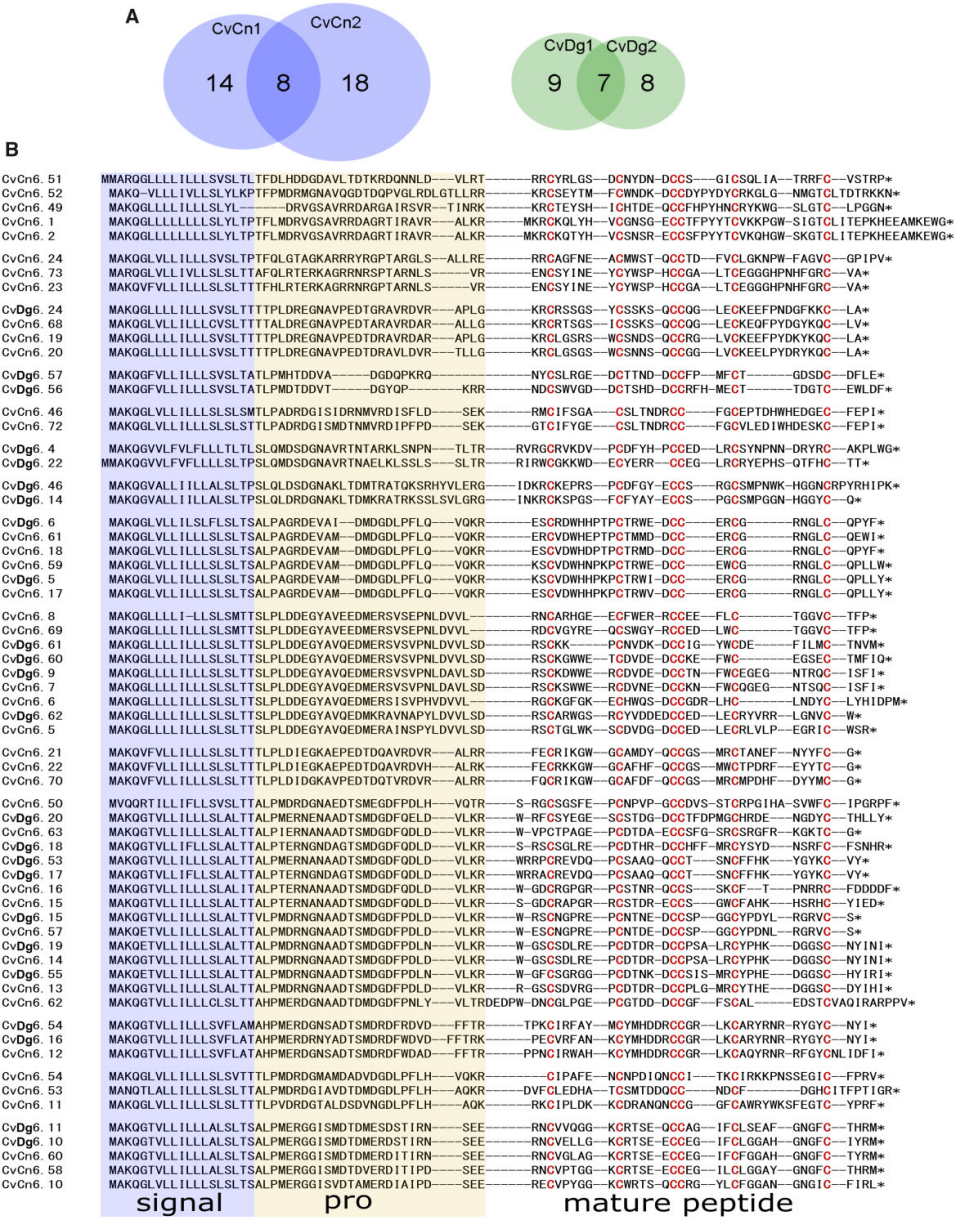
—Diversity of the predicted toxins in the drillipeptide superfamily 7. (*A*) Venn diagram of the drillipeptide diversity in studied specimens of *Clavus*. (*B*) Alignment of 64 predicted complete precursors.

The violin plot (fig. 3*A*) shows distribution of the mature peptide region lengths in the two species of the genus *Clavus* in comparison with published data on some *Conus* species. The pattern of mature peptide region lengths distribution is apparently very close between the two species of *Clavus*. The median length of the mature peptide sequence is at 41 AA for *Cv. canalicularis* and at 44 AA for *Cv. davidgilmouri*, and the lower quartile is at 32 AA in both species. In total, 108 and 58 unique transcripts in the venom gland of *Cv. canalicularis* and *Cv. davidgilmouri*, respectively, show a mature peptide length of <25 AA residues. The putative toxins of 20 gene families with mature toxin regions containing on average 13–30 AA residues closely match in this respect typical conotoxins (figs. 3*B* and 4*B* and supplementary fig. 2, Supplementary Material online). The drillipeptides in the highly diversified superfamily 7, which shows the highest expression level among identified families, are a perfect example of such peptides. For this gene family, a total of 64 individual peptides were predicted from our transcriptomic data (fig. 4*A*), which formed nine orthogroups as inferred from OrthoFinder (supplementary fig. 3, Supplementary Material online). The mature peptide sequences range from 29 to 42 AA residues in length and contain six Cys residues organized in the cysteine framework VI/VII (fig. 4*B*).

The mature toxin regions are on average longer in other lineages of the “Turrid” major clade of conoideans (Puillandre et al. 2011, 2016): *Gemmula speciosa* and *Unedogemmula bisaya* (Turridae), and *Crassispira cerithina* (Pseudomelatomidae) (Gonzales and Saloma 2014). The very limited transcriptomic data on *Gemmula speciosa*, *Unedogemmula bisaya*, and *Crassispira cerithina* make it impossible, however, to test the significance of the difference in mature toxin lengths between Turridae and *Clavus* spp. The broad length range in drillipeptides with typical toxin precursor structure resembles transcriptomes of the Turridae, Pseudomelatomidae, and Terebridae (Imperial et al. 2007; Gorson et al. 2015), whereas the predicted high diversity of relatively short, disulfide-rich peptides in the venom gland transcriptomes of *Clavus* spp. makes them closely comparable with *Conus* venoms. These features of *Clavus* venom gland peptides (high diversity, small size, and predicted stability) suggest that drillipeptides, like conotoxins, may be promising candidates for future pharmacological investigations. Venom peptides with high expression levels can readily be explored by mass spectrometric analysis of *Clavus* venom, followed by chemical synthesis and activity testing. One such bioactive peptide, cdg14a, was identified by mass spectrometry and has been purified from the venom of *Cv. davidgilmouri*. This peptide elicits scratching and hyperactivity followed by a paw-thumping phenotype in mice (Chua et al. forthcoming). The precursor encoding cdg14a, CvDg14.4, from drillipeptide superfamily 7, was found in the transcriptomes of both *Cv. davidgilmouri* specimens (n1-tpm 815, n2-tpm 1,642). Figure 3*C* shows 23 and 22 venom gene superfamilies of *Cv. canalicularis* and *Cv. davidgilmouri* respectively that are both highly expressed (total tpm ≥1,000) and comprise transcripts with mature peptide regions of ≤50 AA (area shown in gray). They could be first analyzed in a search of the most relevant target for subsequent structural studies and bioactivity assays.

Although the majority of predicted peptides in *Clavus* venom share the same canonical structure of signal sequence, propeptide region followed by a single mature toxin with most conotoxin precursors, drillipeptide sequences are quite divergent. We therefore avoid inference of sequence homology between drillipeptides and conotoxins. Nevertheless, as with conotoxins, drillipeptides tend to have an even number of cysteines (typically six) in the mature peptide region. Two of the most common canonical cysteine frameworks for conotoxins, VI/VII (C-C-CC-C-C) and I (CC-C-C), are well represented in the mature peptide regions of drillipeptides (supplementary table 8, Supplementary Material online). However, statistical analysis of the most abundant cysteine frameworks in every category of sequences with an even number of cysteines (4–12) reveals that vicinal cysteines that are common in conotoxin cys-frameworks are not as frequent as scattered single cysteines in drillipeptides (supplementary table 8, Supplementary Material online). Furthermore, the cysteine frameworks V (CC-CC) and III (CC-C-C-CC), each bearing two vicinal cysteines, were only identified in 0 and 2 drillipeptides, respectively despite being ubiquitous in conotoxins.

### Sequence Variability of *Clavus* Insulins Suggests a Role in Predation

Insulin and related peptides (insulin-like growth factors, relaxins, and insulin-like peptides) are peptide hormones that are found throughout the animal kingdom where they play a role in regulating carbohydrate and fat metabolism and as neuromodulators of energy homeostasis and cognition (Smit et al. 1998; Gerozissis and Kyriaki 2003; Blumenthal 2010). Insulin is synthesized as a precursor comprising three regions (A and B chains and a C peptide region), from which proteolytic cleavage of the C peptide in the Golgi releases the insulin heterodimer with A and B chains connected by disulfide bonds. The primary sequence and arrangement of disulfide-forming cysteines are highly conserved in all vertebrates, but invertebrate insulin family members are more variable. Molluskan and most other invertebrate insulins differ from vertebrate insulin in containing two additional cysteines that are assumed to form an additional disulfide between the A and B chain. Moreover, the mature molluskan insulin chains are typically larger than vertebrate insulins. We recently discovered that some species of cone snail weaponized their endogenous insulin for prey capture to induce insulin shock (dangerously low blood glucose levels) in prey (Safavi-Hemami et al. 2015, 2016; Ahorukomeye et al. 2019). *Conus* venom insulins are greatly diversified, specifically expressed in the venom gland, exhibit distinct sequence characteristics from molluskan signaling insulins and correlate with prey taxa (Safavi-Hemami et al. 2016). Cone snail species that prey on mollusks and polychaetes express insulins that closely resemble invertebrate insulins, whereas a subset of fish-hunting species expresses insulins with high sequence and structural similarity to fish insulins which are capable of activating the fish and human insulin receptor (Safavi-Hemami et al. 2015, 2016; Ahorukomeye et al. 2019).

**Fig. 5.**
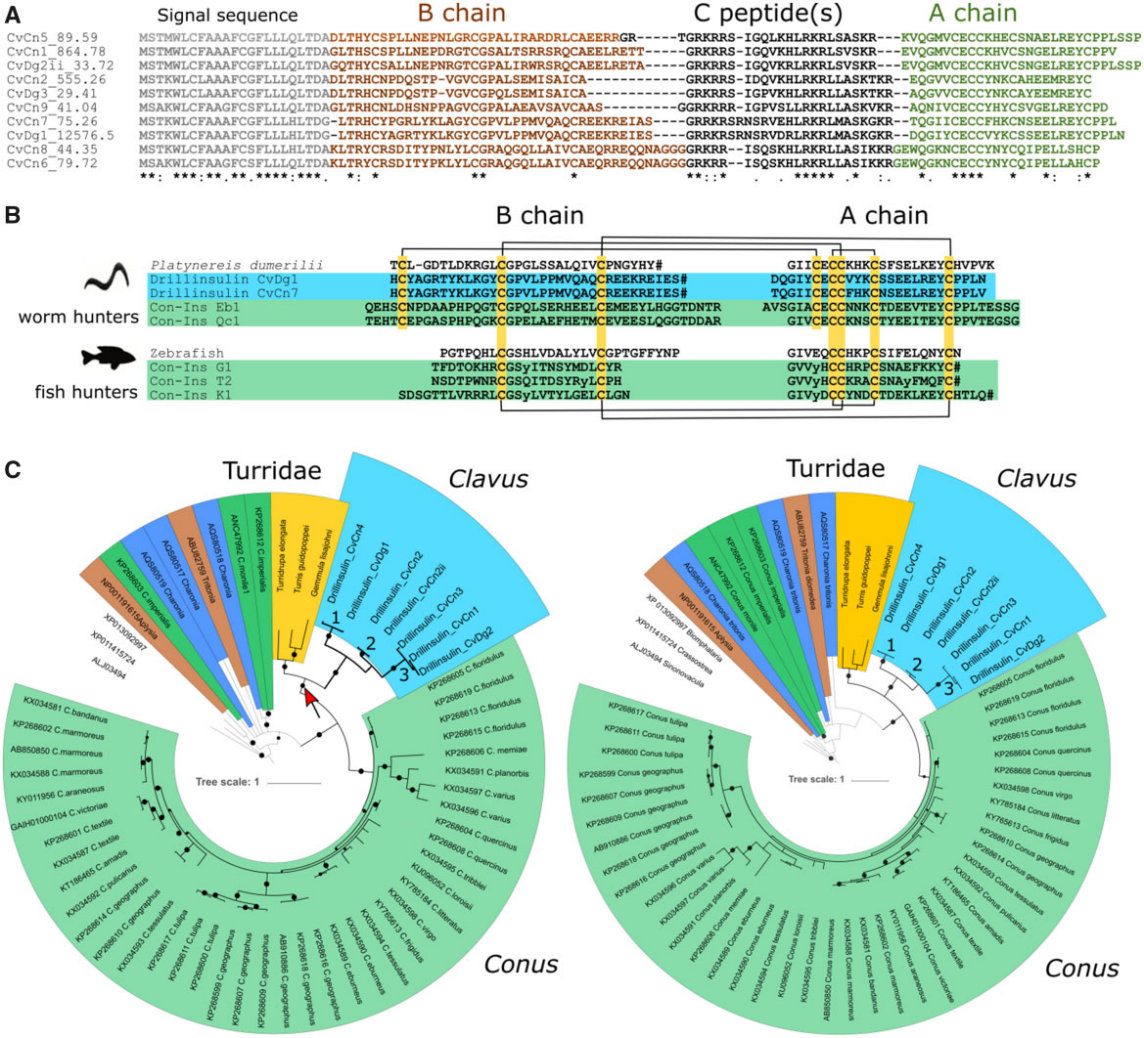
—*Clavus* insulins. (*A*) Comparative sequence alignment of *Clavus* insulins and their expression values (tpm) showing conserved signal peptides and variable A and B chain regions. Amino acid conservations are denoted by an asterisk (*). Full stops (.) and colons (:) represent a low and high degree of similarity, respectively. (*B*) Alignment of the B and A chains of two representative *Clavus* insulins (purple) with insulins sequenced from the marine worm *Platynereis dumerilii* and venom insulins sequenced from worm-hunting cone snails (pink). An alignment of the B and A chains of zebrafish insulin with venom insulins from fish-hunting cone snails is shown for comparison. Cysteines are shown in yellow and disulfide frameworks are depicted as connecting lines. Amino acid code: O = hydroxyproline; y = y-carboxylated glutamate; #, C-terminal amidation. (*C*) Bayesian phylogenetic trees of the gastropod insulins. Left: based on the analysis of entire precursor; right: based on the mature peptide region. Diameter of black circles proportional to support (as posterior probability 0.50–1) of corresponding nodes. Thickened lines denote segment of the tree corresponding to conoidean venom insulins. Colored segments as brown: heterobranchia; dark-blue: *Charonia tritonis* (Tonnoidea); dark green: *Conus* nerve ring; yellow: Turridae; blue: *Clavus* spp.; and light-green: *Conus* venom gland.

Here, we identify seven putative venom insulins in *Clavus* that share several characteristics with *Conus* venom insulins, including significant sequence diversification and high expression levels of some sequences. *Clavus* venom insulins are derived from a single gene superfamily as evidenced by their conserved signal sequence (fig. 5*A*) but exhibit notable sequence divergence within the A and B chains, a hallmark of *Conus* venom insulins (fig. 5*A*). This includes high diversification rates within the primary AA sequences of the A and B chains (apart from a conserved cysteine framework) and changes in chain lengths, most likely resulting from insertion and deletion events (fig. 5*A*). This significant sequence divergence of *Clavus* insulins combined with the very high expression level of drillinsulin CvDg1 in *Cv. davidgilmouri* (n1-tpm 12,576, n2-tpm 3,084) suggests a role of *Clavus* insulins in prey capture. Interestingly, insulins sequenced from *Clavus* share structural similarity with worm and mollusk insulins suggesting that *Clavus* specializes on invertebrate prey as previously reported for snail and worm-hunting *Conus* (Safavi-Hemami et al. 2016) (fig. 5*B*). This is in stark contrast to insulins sequenced from fish-hunting cone snails that exhibit the vertebrate cysteine framework (fig. 5*B*). A more accurate interpretation of these observed patterns will require additional studies to shed light on structure–function properties of the mature venom insulins, their sequence comparison with *Clavus* signaling insulins, and on the spectrum of *Clavus* prey taxa.

Phylogenetic analysis classifies the seven drillinsulins into three clusters (fig. 5*C* left). The cluster 1 drillinsulin CvDg1 shows very high expression levels in *Cv. davidgilmouri* (n1-tpm 12,576, n2-tpm 3,084) and was also detected in *Cv. canalicularis* n2 (drillinsulin CvCn4-tpm 435). Conversely, the cluster 3 insulins (drillinsulin CvCn1-tpm 865, drillinsulin CvCn3-tpm 372) have higher expression in *Cv. canalicularis*, and the cluster 2 insulin is only found in *Cv. canalicularis* (drillinsulin CvCn2-tpm 555, drillinsulin CvCn2ii-tpm 383). Two insulins, drillinsulin CvCn2 (tpm 555) and drillinsulin CvCn2ii (tpm 383) from CvCn1 and CvCn2, respectively, have one AA substitution and likely represent allelic variation of the same locus. In the reconstructed phylogeny of conoidean venom insulin precursors (fig. 5*C* right), the *Clavus* insulins cluster with the insulins of the Turridae species (PP 0.93), and the *Conus* venom insulins form a separate, well supported cluster (PP 1), which reflects phylogenetic relationships among Drilliidae, Turridae, and Conidae (Abdelkrim et al. 2018). In contrast, the phylogenetic analysis of the mature peptide sequence (fig. 5*C* left) does not resolve the relationships among the venom insulins of *Clavus*, Turridae, and *Conus*, although it recovers them in three reciprocally monophyletic clades.

### Molecular Diversity of Porins in *Clavus* Venom

Porins were among the most diversified and highly expressed venom components in all four profiled specimens of *Clavus*. The 25 detected porin transcripts range from 174 to 247 AA in length and share a stretch of about 70 AA in the mature peptide region with high similarity across all sequences (supplementary fig. 4, Supplementary Material online). Nevertheless, these sequence form three clusters distinctive by signal sequence (referred to three separate gene superfamilies, MALLR, MALTL, and MKLFL/MKMFV). Among them, the MALLR sequences show identity to coluporin-12 and coluporin-21 of *Cumia reticulata*. The divergent predicted peptide, here referred to as claviporin-Cn6, shows 32% sequence similarity to tereporin-Ca1 from *Terebra anilis*, and predicted porins in the MALTL gene superfamily display 36–40% sequence similarity to the conoporins from *Conus lividus* and *C. ebraeus*. Therefore, here we consider all three clusters to be porins based on the demonstrated similarity in the mature peptide region (supplementary fig. 4, Supplementary Material online), which also suggests conserved secondary structure. Porins were represented by four to ten unique transcripts per specimen and were typically highly expressed, with a total expression ranging from tpm 4,682 (CvCn1) to tpm 15,072 (CvDg1).

**Fig. 6.**
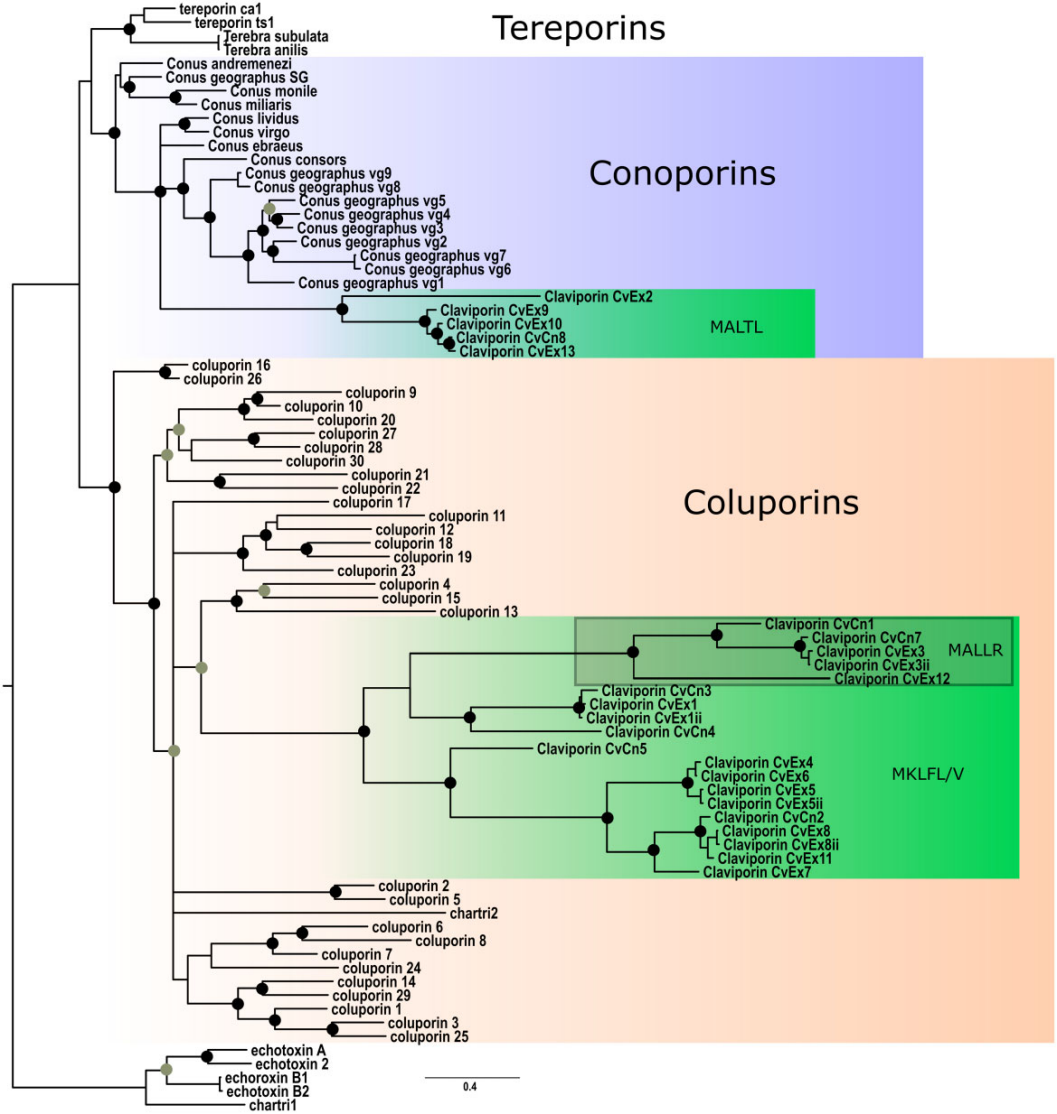
—Bayesian phylogenetic tree of neogastropod porins. Coluporins highlighted with orange, Conoporins—with blue, *Clavus* porin clusters—with green. Nodes with the Bayesian PP support >0.75 marked with gray circles, and with the PP > 0.9—with black circles.

Porins are a class of peptides that bind to and form pores in cellular membranes (Iacovache et al. 2010). Because of their cytolytic effect, porins are involved in host–pathogen interactions, as the agents facilitating infection by a pathogen, as well as a component of the immune response of the host. Porins are a common component of animal venoms, wherein, by disrupting cellular membranes they are thought to facilitate spread of the venom in prey tissues (Parker and Feil 2005). Among marine invertebrates the best studied are the porins of sea anemones (actinoporins) (Álvarez et al. 2009; Rivera-de-Torre et al. 2016; Podobnik and Anderluh 2017), and in gastropods—the echotoxins of tonnoideans (Kawashima et al. 2003; Bose et al. 2017) and coluporins of the Mediterranean bloodsucking neogastropod *Cumia reticulata* (Gerdol et al. 2018). In *Cumia*, documented notable expansion of the porin gene superfamily is explained by their functional importance for penetrating prey circulatory system, and by their hemolytic effect. Despite the suggestion of Gerdol et al. (2018) that porins are only present in two conoidean lineages, cone snails (in particular *Conus geographus*—Safavi-Hemami et al. 2014), and the family Terebridae (Gorson et al. 2015), our results demonstrate that porins are not only present but also highly diversified and expressed in *Clavus*, and, possibly, in other drilliids. Furthermore, the phylogenetic analysis of the *Clavus* porins with other neogastropod porins (Gerdol et al. 2018) revealed that they actually fall into two separate clusters (fig. 6). The MALTL superfamily groups with the conoporins, whereas the remaining two clusters form a clade nested in the coluporins, and both relationships are highly supported. This result has several important implications. Porins of *Cumia* were shown to be divergent from all previously known porins of Conoidea (Gerdol et al. 2018), mirroring distant relationship between Conoidea and Buccinoidea. Such concordance of the species tree and the gene tree recovered for one or another gene superfamily characterized from transcriptomic data is a predominant pattern in studies of gastropod venoms (e.g., Terebridae—Gorson et al. 2015, *Cumia*—Gerdol et al. 2018, and *Profundiconus*—Fassio et al. 2019). The contrasting example is the relationships of *Conus* insulins, which reflects the relationship among the prey taxa (Safavi-Hemami et al. 2016). The phylogeny of *Clavus* porins, however, shows a third pattern, which is different from the two mentioned above. It is consistent with porins having been recruited from two separate gene superfamilies: One presumably was present in the common ancestor of Conoidea and *Cumia* (but never previously detected in Conoidea), and the other one, on the contrary, only currently known in Conoidea. The presence of products of both these gene clusters in *Clavus* may suggest that their molecular targets (e.g., in vertebrate vs. invertebrate systems) and/or action mechanisms differ from one another. This hypothesis is consistent, for example, with the distinction between the defensive and predatory porins. Further studies on the diversity of porins in Neogastropoda, and on the spatial differentiation of gene expression in the *Clavus* venom gland, and the spectrum of molecular activities are necessary to better understand diversity and evolution of venom porins in the Neogastropoda.

### Structural Diversity of RBLs

Carbohydrate-binding proteins, lectins, are ubiquitous and diverse in nature. RBLs, which play roles in innate immunity in fish (Watanabe et al. 2009), formed one of the remarkably diverse clusters of the predicted venom components of *Clavus* spp. Six predicted *Clavus* venom transcripts with expression levels above 100 tpm showed detectable similarity to the l-RBLs of the Chum salmon *Oncorhynchus keta* and the Amur catfish *Silurus asotus*. When the transcripts with lower expression levels were considered, a total of 28 transcripts encoding RBLs were identified, their tpm expression levels ranging from 5 to 15,382. The majority (24 transcripts) of the predicted RBL precursors comprised two carbohydrate recognition domains, (CRDs) (as defined by Ogawa et al., 2011), in which, it is interesting that the two CRDs of the same sequence have the same number of cysteines. The alignment of the CRDs is provided in supplementary figure 5, Supplementary Material online, with the secondary structure predictions mainly based on the annotation provided by Ogawa et al. (2011). Although the CRDs from Drillilectin_CvCn1, the most abundant RBL from *Cv. canalicularis* (indicated in supplementary fig. 5, Supplementary Material online, by green arrows, tpm: 1,542.16) has the canonical eight cysteines to form four disulfide bonds, the CRDs from Drillilectin_CvDg1, the most abundant RBL from *Cv. davidgilmouri* (indicated with red arrows, tpm: 15,382.68) has six cysteines and a short sequence lacking the C1–C3 disulfide bond. In addition, one predicted RBL transcript (Drillilectin_CvCn11, highlighted with orange in supplementary fig. 5, Supplementary Material online) comprised three CRDs and three transcripts (Drillilectin_CvDg3, Drillilectin_CvCn5, and Drillilectin_CvDg10, highlighted with purple) comprised four CRDs. All CRDs in Drillilectin_CvCn11, Drillilectin_CvCn5, and Drillilectin_CvDg3 have only three disulfide bonds, but all CRDs in Drillilectin_CvDg10 have four. The CRDs in *Clavus* RBLs typically contain the “YGR” and “DPC” motifs characteristic for other RBLs identified from marine animals (e.g., sea anemone, urchin, oyster, or fish), despite the overall sequence identity of the *Clavus* RBLs to previously identified RBLs being rather low (on average <=30%). The high diversity and in some cases very high expression levels of lectins suggest that they may play an important role in envenomation. This hypothesis for Conoidea in general is supported by the evidence of high RBL differential expression in the venom gland of *Profundiconus vaubani*, the most early divergent cone snail genus, and a distant conoidean relative of *Clavus* (Fassio et al. 2019).

Both lectins and porins bind to the components of cellular membranes, and both are involved in immunity, due to their demonstrated ability to disrupt cellular membranes (Parker and Feil 2005; Watanabe et al. 2009). The high diversity and expression levels of both these protein classes in the venom of *Clavus* can possibly be explained by their action, which may facilitate venom penetration and spreading in the prey body (consistent with the hypothesis of Gerdol et al on *Cumia*). The radula of *Clavus*, with its flat marginals and comb-like lateral teeth, cannot be used to inject venom into the prey’s body, as in *Conus*. However, based on the anatomical studies, drilliids use marginal teeth in a similar manner—holding a tooth at the tip of the proboscis presumably to make lacerations on the prey’s integument (Sysoev and Kantor 1989). Through the laceration venom can penetrate the prey’s body, but to cause major effects on the prey’s physiological circuits, it should reach and be spread by the circulatory system. This local diffusion of venom can be faster if adjacent epithelia are damaged by, for example, cytolytic polypeptides. Thus, the high abundance and diversity of lectins and porins—proteins with demonstrated cytolytic activity—could be a molecular adaptation of *Clavus* in the lack of efficient mechanical venom delivery achieved in *Conus* by means of hypodermic radular teeth.

## Conclusions

The venom gland transcriptomes of previously unstudied predatory gastropods of the genus *Clavus* demonstrate unique biochemical features: displaying a diversity of gene families unparalleled among conoideans, most of which do not show similarity to known toxin families. Despite the phylogenetic position of *Clavus* in the “turrid” major clade of the Conoidea (that diverged from the “conid” major clade, including the genus *Conus* around 130 Ma—Abdelkrim et al. 2018), some putative *Clavus* venom peptides show detectable similarity to conotoxins. Furthermore, our data suggest that the venoms of *Clavus* are dominated by rather short peptides, and in this regard resemble more the venom of *Conus* than venoms of more closely related Turridae and Terebridae. The demonstrated high diversity of short cysteine-rich peptides encoded in the venom gland transcriptomes of *Clavus* suggests they may also represent a valuable resource for drug discovery. Future studies on the characterization of the physiological effects and the identification of molecular targets of these peptides will elucidate the pharmacological potential of drillipeptides. Alongside the short cysteine-rich peptides, the venom of *Clavus* is remarkable for the high diversity and expression levels of insulins, RBLs, and porins. Insulins are likely to disrupt energy metabolism of prey, whereas porins and lectins may exert cytolytic effects that enhance the spread of venom in the prey’s body. This is the first convincing evidence of complex biochemical adaptation leveraging the lack of efficient venom delivery apparatus in the “turrid” lineage of Conoidea. Furthermore, it is certain that venom insulins, lectins, and two gene families of porins were already present in the common ancestor of *Conus* and *Clavus*—that is, in one of the earliest conoideans. Conversely, the available data are not sufficient to infer whether some known conotoxin gene superfamilies have homologs in the transcriptome of *Clavus*. Thus, we reveal a novel, and in many aspects, a unique fragment of knowledge of conoidean venom evolution, which will likely inspire future studies on this remarkable, yet still poorly studied taxon.

## Supplementary Material

evaa083_Supplementary_Data

## Notes

**Data deposition:** Raw sequence data analyzed in this article have been deposited at NCBI under the accession PRJNA610292.
