# Supplementary material for: Transcriptomic Profiling Reveals Extraordinary Diversity of Venom Peptides in Unexplored Predatory Gastropods of the Genus Clavus
Source: Genome Biol Evol. 2020 Apr 23;12(5):684–700. doi: 10.1093/gbe/evaa083 (PMC7259678; doi:10.1093/gbe/evaa083)
Supplement: evaa083_Supplementary_Data [file evaa083_supplementary_data.zip › Clavus_Supplementary_figures.pdf]

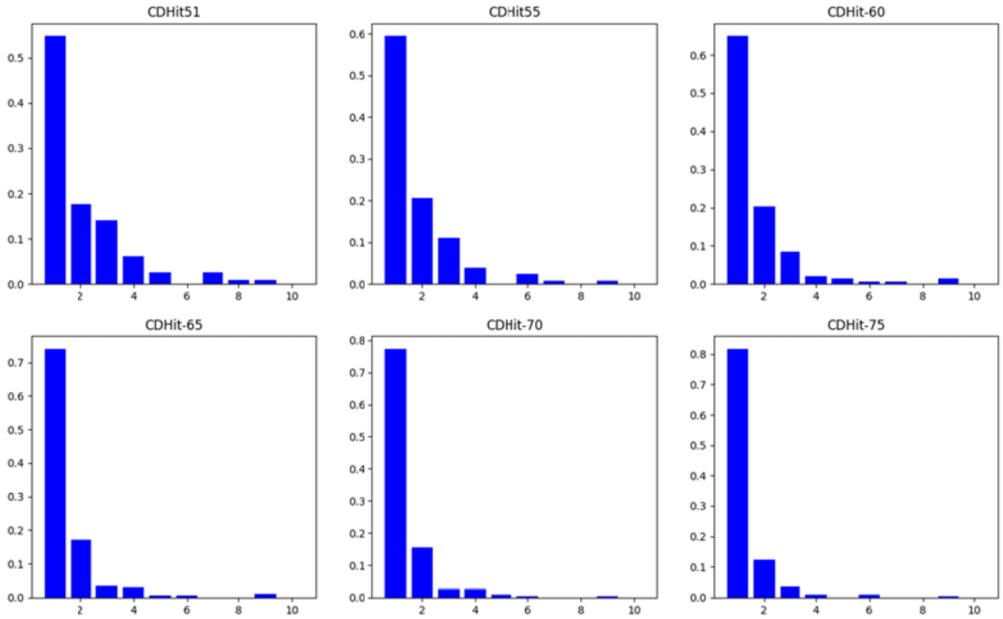

Supplementary Figure 1. Number of different Cys-patterns per cluster in different CD-Hit cluster sets.

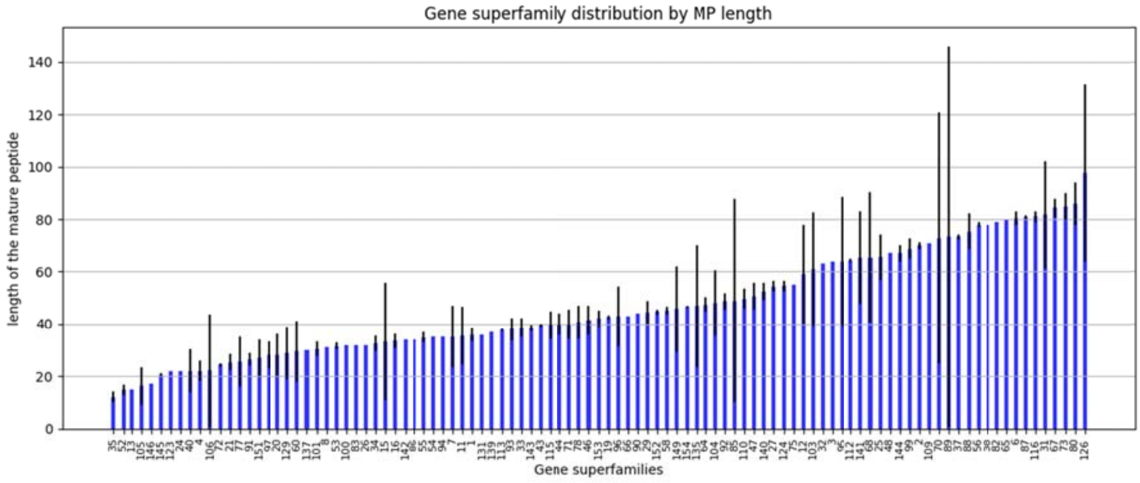

Supplementary Figure 2. Gene suprfamilies distribution by the mature peptide region length.

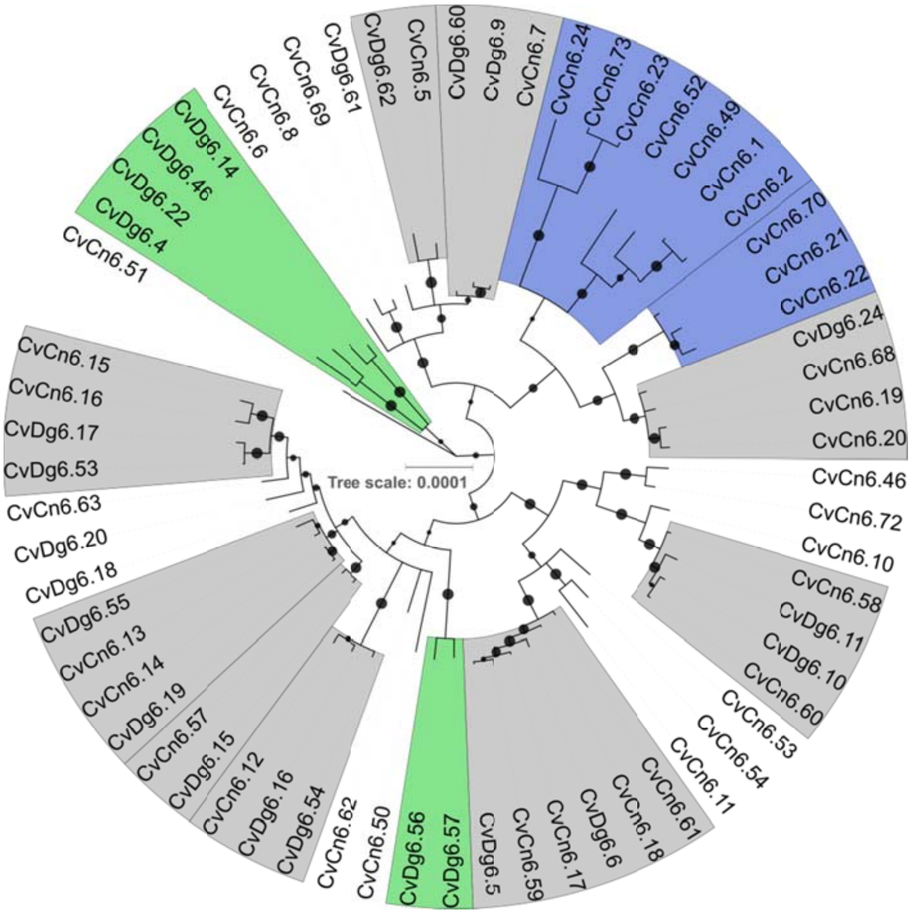

Supplementary Figure 3. Bayesian phylogenetic tree of the 64 predicted gene superfamily 4 toxins identified in *Clavus*. Blue segments correspond to venom components solely found in *Cv. canalicularis*, green – in *Cv. davidgilmouri*, grey – orthogroups, identified by OrthoFinder including transcripts from both species. Diameter of black circles proportional to support (as Posterior probability 0.50 – 1) of corresponding nodes.

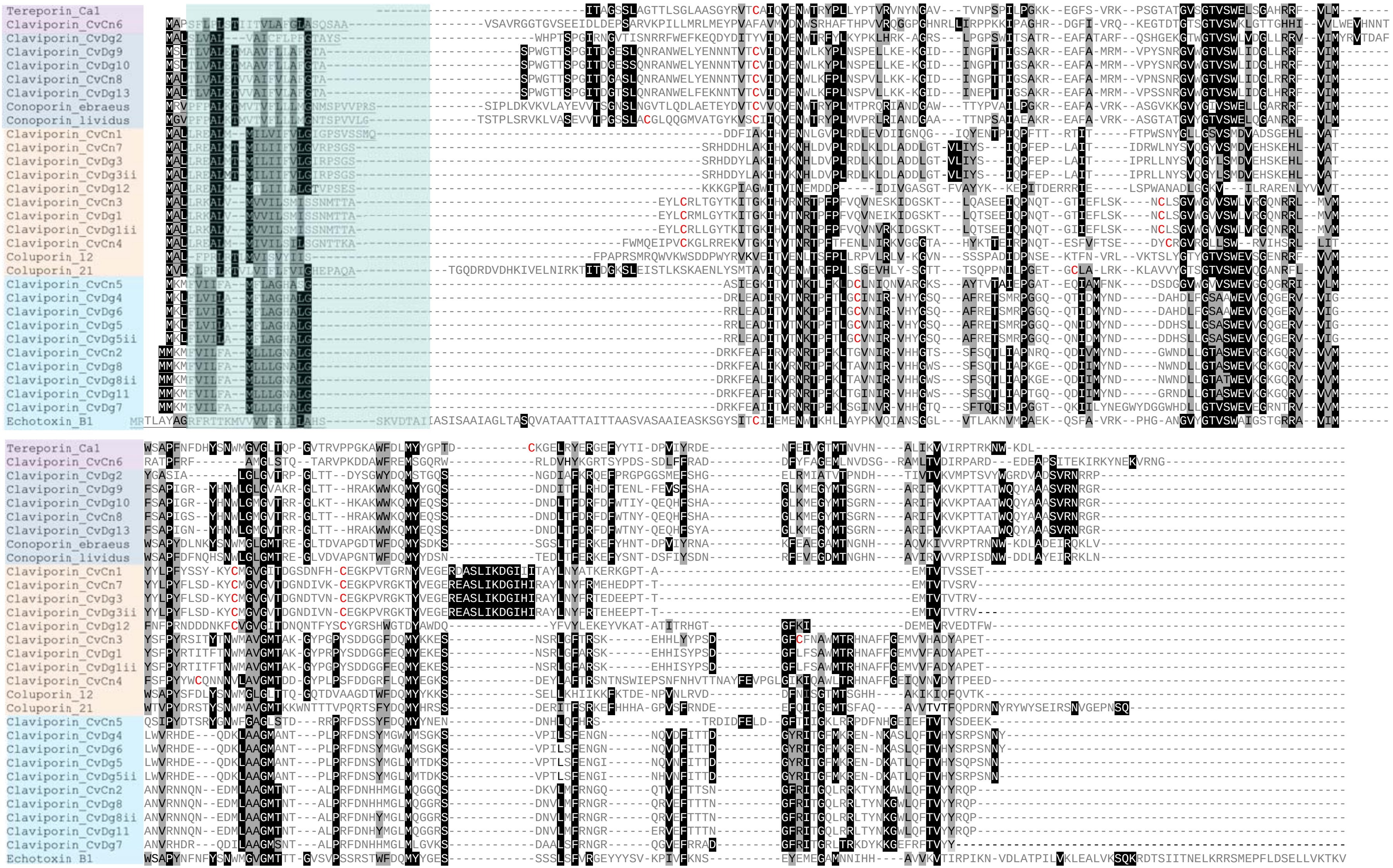

Supplementary Figure 4. Alignment of the *Clavus* porins with some porins identified in other taxa of predatory gastropods. Three clusters of porins (plus claviporin-Cv6 separately) showed with different color shading. Signal and pro- regions highlighted and underlined.
