# Supplementary material for: Transcriptomic Profiling Reveals Extraordinary Diversity of Venom Peptides in Unexplored Predatory Gastropods of the Genus Clavus
Source: Genome Biol Evol. 2020 Apr 23;12(5):684–700. doi: 10.1093/gbe/evaa083 (PMC7259678; doi:10.1093/gbe/evaa083)
Supplement: evaa083_Supplementary_Data [file evaa083_supplementary_data.zip › Clavus_Supplementary_tables.docx]

Supplementary Table1. RNAseq data sets used for this analysis. The number of reads, read length and quality were calculated for samples before and after quality/adaptor trimming.

| *Specimen* | Before quality/adaptor trimming | | | After quality/adaptor trimming | | |
| --- | --- | --- | --- | --- | --- | --- |
|  | Number of reads | Read length(nt) | Medium  quality | Number of reads | Read length(nt) | Medium  quality |
| *Clavus davidgilmouri* 1 | 26,270,594 | 125 | 32 | 25,091,770 | 80-125 | 37 |
| *Clavus davidgilmouri* 2 | 70,929,006 | 125 | 30 | 67,744,294 | 80-125 | 37 |
| *Clavus canalicularis* 1 | 46,545,640 | 125 | 32 | 44,455,740 | 80-125 | 37 |
| *Clavus canalicularis* 2 | 50,114,446 | 125 | 32 | 47,864,308 | 85-125 | 37 |

Supplementary Table 2. Summary of assembly and annotation statistics for the 4 RNAseq data sets.

| Specimen | N50 (bp) | Total assembly size (bp) | Total number of contigs | Shortest Contig (nt) | Longest Contig (nt) |
| --- | --- | --- | --- | --- | --- |
| *Clavus davidgilmouri* 1 | 664 | 5,233,597 | 9,580 | 201 | 10,301 |
| *Clavus davidgilmouri* 2 | 311 | 6,258,470 | 18,933 | 201 | 4,911 |
| *Clavus canalicularis* 1 | 573 | 8,848,576 | 18,009 | 201 | 7,156 |
| *Clavus canalicularis* 2 | 493 | 7,721,636 | 17,250 | 201 | 6,746 |

Supplementary Table 3. Statistics of the tested cluster sets.

| Sig-sequence identity threshold | Number of CD-Hit clusters | Score against Blast-based clusters | Penalty against OrthoFinder (orthogroups split between 2 or more gene superfamilies) |
| --- | --- | --- | --- |
| 51 | 120 | 0.771428571429 | -36 |
| 55 | 136 | 0.761904761905 | -34 |
| **60** | **158** | **0.771428571429** | **-23** |
| 65 | 189 | 0.647619047619 | -23 |
| 70 | 221 | 0.552380952381 | -30 |
| 75 | 245 | 0.552380952381 | -31 |

Supplementary Table 4. Numbers of identical clusters in pairwise comparison of the inferred cluster sets.

|  | BLAST | CD51 | CD55 | CD60 | CD65 | CD70 | CD75 |
| --- | --- | --- | --- | --- | --- | --- | --- |
| BLAST |  |  |  |  |  |  |  |
| CD51 | 80 |  |  |  |  |  |  |
| CD55 | 79 | 87 |  |  |  |  |  |
| **CD60** | **83** | **74** | **95** |  |  |  |  |
| CD65 | 79 | 63 | 81 | **122** |  |  |  |
| CD70 | 76 | 56 | 71 | **100** | 149 |  |  |
| CD75 | 71 | 54 | 67 | **92** | 134 | 181 |  |

Supplementary Table 5. Pearson’s correlation coefficients for the intra- and interspecific comparisons

1. **Superfamily unique transcript numbers:**

CvCn 1 - CvCn 2 Pearson.s R: (0.893, 4.68e-56)

CvDg 1 - CvDg 2 Pearson.s R: (0.858, 4.55e-47)

mean CvCn - mean CvDg Pearson.s R: (0.829, 2.71e-41)

1. **Superfamily expression levels:**

CvCn 1 - CvCn 2 Pearson’s R: (0.622, 2.63e-18)

CvDg 1 - CvDg 2 Pearson’s R: (0.815, 8.35e-39)

mean CvCn - mean CvDg Pearson’s R: (0.617, 6.13e-18)

Supplementary Table 6. Shannon’s diversity index

Specimen Shannon’s index (H’) Evenness (E)

CvCn1 4.24036150227 0.895309085646

CvCn2 4.26682816545 0.899239030195

CvDg1 4.47425164396 0.926669617467

CvDg2 4.32592494736 0.927625484123

Supplementary Table 7. Net-between groups distance for three clusters of A. *Clavus* venom insulins, and B. *Conus geographus* venom insulins.

| **A** | Clav1 | Clav2 | Clav3 |  | **B** | geogr-m | geogr-f1 | geogr-f2 |
| --- | --- | --- | --- | --- | --- | --- | --- | --- |
| Clav1 |  |  |  |  | geogr-m |  |  |  |
| Clav2 | 0.974 |  |  |  | geogr-f1 | 1.748 |  |  |
| Clav3 | 1.470 | 0.739 |  |  | geogr-f2 | 1.68 | 0.519 |  |

Supplementary Table 8. Single-scattered cysteine frameworks are preferred in drillipeptides. Occupancy of some canonical cysteine frameworks in conotoxin-like drillipeptide shown in Figure 5 are also listed.

| Cysteine Framework | Number (Occupancy) | tpm (Occupancy) |
| --- | --- | --- |
| All patterns with 4 cysteine (n=86, tpm=97624) | | |
| CC-C-C | 7 (8.1%) | 3822 (3.9%) |
| C-C-C-C | 71 (82.6%) | 89360 (91.5%) |
| All patterns with 6 cysteines (n=453, tpm=427045) | | |
| C-C-CC-C-C | 209 (46.1%) | 171383 (40.1%) |
| C-C-C-C-C-C | 199 (43.9%) | 227733 (53.3%) |
| All patterns with 8 cysteines (n=215, tpm=197939) | | |
| C-C-CC-C-C-CC | 10 (4.7%) | 4414 (2.22%) |
| C-C-CC-C-C-C-C | 37 (17.2%) | 18022 (9.1%) |
| C-C-C-C-C-C-C-C | 142 (66%) | 162257 (82%) |
| All patterns with 10 cysteines (n=71, tpm=84436) | | |
| C-C-C-C-C-C-C-C-C-C | 37 (52.1%) | 69279 (82%) |
| All patterns with 12 cysteines (n=63, tpm=48727) | | |
| C-C-C-C-C-C-C-C-C-C-C-C | 47 (74.6%) | 34482 (70.8%) |
